# Genomic and functional insights into antibiotic resistance genes *floR* and *strA* linked with the SXT element of *Vibrio cholerae* non-O1/non-O139

**DOI:** 10.1099/mic.0.001424

**Published:** 2024-01-05

**Authors:** Mousumi Saha, Agila Kumari Pragasam, Shashi Kumari, Jyoti Verma, Bhabatosh Das, Rupak K. Bhadra

**Affiliations:** ^1^​ Infectious Diseases and Immunology Division, Indian Institute of Chemical Biology (CSIR), Kolkata-700032, India; ^2^​ Centre for Microbial Research, Translational Health Science and Technology Institute, Faridabad-121001, Haryana, India

**Keywords:** antibiotic resistance, cholera, SXT, genome

## Abstract

The emergence and spread of antibiotic-resistant bacterial pathogens are a critical public health concern across the globe. Mobile genetic elements (MGEs) play an important role in the horizontal acquisition of antimicrobial resistance genes (ARGs) in bacteria. In this study, we have decoded the whole genome sequences of multidrug-resistant *Vibrio cholerae* clinical isolates carrying the ARG-linked SXT, an integrative and conjugative element, in their large chromosomes. As in others, the SXT element has been found integrated into the 5′-end of the *prfC* gene (which encodes peptide chain release factor 3 involved in translational regulation) on the large chromosome of *V. cholerae* non-O1/non-O139 strains. Further, we demonstrate the functionality of SXT-linked *floR* and *strAB* genes, which confer resistance to chloramphenicol and streptomycin, respectively. The *floR* gene-encoded protein FloR belongs to the major facilitator superfamily efflux transporter containing 12 transmembrane domains (TMDs). Deletion analysis confirmed that even a single TMD of FloR is critical for the export function of chloramphenicol. The *floR* gene has two putative promoters, P1 and P2. Sequential deletions reveal that P2 is responsible for the expression of the *floR*. Deletion analysis of the N- and/or C-terminal coding regions of *strA* established their importance for conferring resistance against streptomycin. Interestingly, qPCR analysis of the *floR* and *strA* genes indicated that both of the genes are constitutively expressed in *V. cholerae* cells. Further, whole genome-based global phylogeography confirmed the presence of the integrative and conjugative element SXT in non-O1/non-O139 strains despite being non-multidrug resistant by lacking antimicrobial resistance (AMR) gene cassettes, which needs monitoring.

## Introduction

*Vibrio cholerae*, the causative agent of the severe diarrhoeal disease cholera, is a Gram-negative, motile γ-proteobacterium. Currently, there are more than 200 reported serogroups of *V. cholerae* [1]. Not all *V. cholerae* strains are pathogenic; only the strains belonging to the serogroups O1 and O139 are responsible for cholera pandemics [2]. Strains belonging to serogroups other than O1/O139 are collectively called non-O1/non-O139, and are usually non-pathogenic but may cause sporadic ‘cholera-like’ diarrhoea in humans. When pathogenic *V. cholerae* cells are ingested by a human host, through contaminated food or water, they travel through the acidic stomach, reach the intestine, and ultimately colonize in the small intestine using the toxin co-regulated pilus (encoded by the *tcpA* gene), and produce the potent enterotoxin cholera toxin, which is responsible for the massive secretory diarrhoea. Interestingly, the genome of *V. cholerae* is composed of two chromosomes, one large (~3.0 Mb) and one small (~1.1 Mb). Several genetic studies have established that the organism is proficient at recombination and may exchange various virulence and antibiotic resistance (AMR) genes linked with mobile genetic elements (MGEs) through horizontal gene transfer (HGT). An MGE, such as the integrative and conjugative element (ICE) SXT, is self-transmissible, may integrate into chromosome, and can provide drug resistance against different antibiotics, such as sulfamethoxazole, trimethoprim, streptomycin and several others [3]. ICEs are increasingly recognized as important mediators of HGT among prokaryotes, and they play important roles in the evolution of variants through the expression of their genes, which may help the recipient cells adapt to various stressful environmental conditions [4].

The genomes of *V. cholerae* non-O1/non-O139 contain a variety of MGEs, including AMR-encoding genes (ARGs); however, little is known about their regulation and expression. It is believed that extensive genetic exchange between different microbes in the environment as well as in the host-associated state may lead to the genesis of drug-resistant strains. The genes that confirm AMR functions in *V. cholerae* are highly heterogeneous and mostly linked with MGEs [5, 6]. Major outer membrane porin proteins (OMPs) of *V. cholerae*, namely OmpU and OmpT, may confer resistance to different xenobiotics, including antibiotics [7]. Many of the antibiotic efflux pumps identified in *V. cholerae* belong to the multidrug and toxic compound extrusion (MATE) and resistance nodulation division (RND) families of efflux proteins and exhibit particularly broad substrate specificity [8].

The SXT element was first identified in *V. cholerae* O139 strains isolated in Madras (now Chennai), India in 1992 [3]. The SXT is a 100 kb long integrative genetic element. The genes carried by the SXT element usually code for resistance to chloramphenicol (*floR*), streptomycin (*strAB*), sulphamethoxazole (*sulII*), trimethoprim (*dfrA1* and *dfr18*), etc. [3, 9–11]. SXT integrates into the 5′-end of *prfC*, a gene encoding peptide chain release factor 3 found in the large chromosome of *V. cholerae* [10]. Integration and excision of the SXT element from the chromosome require an SXT-encoded tyrosine recombinase, which belongs to the family of site-specific tyrosine recombinases [4]. The SXT element (also known as ICE*Vch*Ind4) appears to be important in the evolution of *V. cholerae* O1 and O139 serovars. Abrupt loss of the SXT element with the gene cassette containing *floR*, *strAB*, *sul2* and *dfrA18* was found to be one the reasons for the epidemiological decline of O139 serogroup [12].

Florfenicol is a fluorinated chloramphenicol derivative used as a clinical veterinary drug [13, 14]. Resistance to it has been detected in a wide variety of enteric bacteria, including *Escherichia coli*, *Klebsiella pneumoniae*, *Salmonella enterica*, *V. cholerae,* etc. The *floR* gene is responsible for conferring resistance against chloramphenicol and its analogues [15]. While in *V. cholerae* and other bacteria the *floR* gene is present in the chromosome, in *K. pneumoniae* it is carried by a plasmid [16]. However, in the case of *E. coli*, the *floR* could either be present on the chromosome or on a plasmid [17]. The *floR* encodes FloR, which is an efflux pump protein consisting of 12 TMDs and belongs to the DHA12 (drug: H^+^ antiporter of 12 spanners) family of the major facilitator superfamily (MFS) [18–20]. Most of the functionally active FloR is ~400 amino acids long. FloR is a transporter specific for structurally associated phenicol drugs that utilizes the proton motive force (PMF) to energize an active efflux mechanism to pump specific drug outside of a cell by a PMF-driven mechanism. Charged amino acids play a vital role in the activity of this efflux pump. Negatively charged amino acids located at TM number 1 (TM1) are probably necessary for chloramphenicol, thiamphenicol and florfenicol diffusion [21]. They are also partly involved in the ligand recognition pocket of the pump and in the specificity of florfenicol transport. Arginine in TM number 4 (TM4) is a highly conserved residue. This residue preserves a significant chloramphenicol efflux, and the resistance level is partially conserved. Amino acids in TM number 9 (TM9) are important for membrane assembly and pump stability. They are probably involved in the folding of the FloR pump [21]. However, currently no study has been conducted to determine precisely which domains of the FloR protein are essential for efflux pump activity. Furthermore, Karlsson *et al.* [22] have shown intermediate susceptibility to chloramphenicol in *V. cholerae* O1 strains carrying the *floR*, which could be due to its low expression. Thus, there is a need to know the regulation of expression of ARGs, which may help in the future to overcome the problem related to multidrug-resistant bacterial strains.

The current study aims to (i) map the chromosomal integration sites of the SXT element, including the *int*, in the genome of the multidrug-resistant clinical *V. cholerae* non-O1/non-O139 strain IDH07118, (ii) study whole genome-based global phylogeography for SXT positive non-O1/non-O139 strains, and (iii) perform a mutational and functional analysis of the *strAB* and *floR* genes present in the SXT element, which confer streptomycin and chloramphenicol resistance, respectively, to the IDH07118 strain.

## Methods

### Bacterial strains, plasmids and growth conditions

The bacterial strains and plasmids used in this study are listed in Table 1. Bacterial strains were usually grown in Luria broth (LB; Difco, USA) at 37 °C with shaking and with Luria agar (LA; Difco) for plate culture. For transformation, *E. coli* DH5α (Table 1) strain was used. Bacterial strains were preserved at −70 °C in LB containing 20 % sterile glycerol. Antibiotics (Sigma-Aldrich, USA) were used at the following concentrations unless mentioned otherwise: ampicillin (Amp), 100 µg ml^−1^; kanamycin (Kan), 40 µg ml^−1^; streptomycin (Sm), 100 µg ml^−1^; and chloramphenicol (Cam), 3 µg ml^−1^ for *V. cholerae.* Before initiation of any experiment, bacterial strains were freshly inoculated from their −70 °C stock. Bacterial growth was monitored by measuring optical density (OD) at 600 nm (OD_600_) using a spectrophotometer (Model U-5100; Hitachi, Japan).

**Table 1.** Bacterial strains and plasmids used in this study

Strain	Relevant genotype and/or phenotype	Source/reference
***V. cholerae***		
N16961	WT clinical O1 El Tor strain, lacking *hapR* function, Sm^r^	[47]
C6709	WT clinical O1 strain, with *hapR* function, Sm^r^	[48]
FGL9582	WT clinical O1 El Tor strain	T. Ramamurthy
IDH07118	WT clinical non-O1/non-O139 strain, Cam^r^, Sm^r^	T. Ramamurthy
FGL6615	WT clinical non-O1/non-O139 strain	T. Ramamurthy
FGL7710	WT clinical non-O1/non-O139 strain	T. Ramamurthy
SG24	WT clinical O139 strain, Cam^r^, Sm^r^	[11]
VCE232	WT environmental strain of O4 serogroup	Lab stock
***E. coli***		
DH5α	F’ *endA1 hsdR17 supE44 thi-1 recA1 gyrA96 relA1* Δ(*argF-lacZYA*) *U169* (Φ80d*lacZ*ΔM15)	Lab stock
**Plasmids**		
pDrive	pUC origin, high-copy-number cloning vector; Amp^r^Kan^r^	QIAGEN
pBAD24	pBR322 origin, l-arabinose inducible expression vector; Amp^r^	Labstock
pDFloR	1.9 kb of the *floR*gene with its promoter of the strain IDH07118 cloned in *Eco*RI/*Pst*I digested pDrive; Amp^r^, Kan^r^, Cam^r^	This study
pDFloRΔP1	1.58 kb of the *floR* gene with its truncated promoter region of the IDH07118 strain cloned in *Eco*RI/*Pst*I digested pDrive; Amp^r^, Kan^r^	This study
pDFloRΔP1P2	1.47 kb of the *floR* gene with its truncated promoter region of the IDH07118 strain cloned in *Eco*RI/*Pst*I digested pDrive; Amp^r^, Kan^r^	This study
pFloRORFBAD	1.22 kb of the *floR* ORF of the strain IDH07118 cloned in *Eco*RI/*Pst*I digested pBAD24; Amp^r^,Cam^r^	This study
pFloRNΔ29BAD	pFloRORFBAD with 87 bp deletion from 5’-end of *floR* ORF; Amp^r^	This study
pFloRNΔ4BAD	pFloRORFBAD with 12 bp deletion from 5’-end of *floR* ORF; Amp^r^	This study
pFloRCΔ36BAD	pFloRORFBAD with 108 bp deletion from 3’-end of *floR* ORF; Amp^r^	This study
pFloRCΔ12BAD	pFloRORFBAD with 36 bp deletion from 3’-end of *floR* ORF; Amp^r^	This study
pFloRNΔ4CΔ12BAD	pFloRNΔ4BAD with 36 bp deletion from 3’-end of *floR* ORF; Amp^r^	This study
pStrABBAD	1.65 kb *strAB* ORF of IDH07118 cloned in *Eco*RI/*Pst*I digested pBAD24; Amp^r^, Sm^r^	This study
pStrABAD	0.8 kb *strA* ORF of IDH07118 cloned in *Eco*RI/*Pst*I digested pBAD24; Amp^r^, Sm^r^	This study
pStrBBAD	0.85 kb *strB* ORF of IDH07118 cloned in *Eco*RI/*Pst*I digested pBAD24; Amp^r^, Sm^r^	This study
pStrANΔ27BAD	pStrABAD with 81 bp deletion from 5’-end of *strA* ORF; Amp^r^	This study
pStrACΔ6BAD	pStrABAD with 18 bp deletion from 3’-end of *strA* ORF; Amp^r^	This study

FGL, Functional Genomics Laboratory; IDH, Infectious Diseases Hospital; ORF, Open reading frame; WT, Wild type; Δ, Deletion.

### Antimicrobial susceptibility testing

Bacterial strains and those carrying the recombinant plasmids were tested for their susceptibility to various concentrations of chloramphenicol (3–5 µg ml^−1^) and streptomycin (100–200 µg ml^−1^) using the broth dilution method. The entire assay was performed with relevant sensitive control strains of *V. cholerae*.

### Molecular biological methods

Standard molecular biological methods [23] for chromosomal and plasmid DNA preparations, restriction enzyme digestion, DNA ligation, bacterial transformation, electroporation, agarose gel electrophoresis, etc*.,* were followed unless stated otherwise. Restriction and nucleic acid-modifying enzymes were purchased from New England Biolabs, Inc. (NEB, USA) and were used essentially as directed by the manufacturer. T4 DNA ligase enzyme used in this study was procured from Promega (USA). Ligation reactions were carried out essentially as directed by the manufacturer. Electrocompetent *V. cholerae* cells were prepared essentially as reported earlier [24] and transformants were selected on LA plates containing appropriate antibiotics.

### PCR assays

To detect the SXT integrase gene in the *V. cholerae* IDH07118 strain (Table 1), the primers SXTint-F/SXTint-R were used (Table 2). The primers SIint-F/SIint-R (Table 2) were used to detect the superintegron (SI) site-specific recombinase gene (*intI4*) in the strain IDH07118. PCR of the ARG conferring resistance to chloramphenicol (*floR*) was done using the primer pair FloRfl-F/FloRfl-R (Table 2) and the genomic DNA of the IDH07118 strain. Genomic DNA of *V. cholerae* serogroup O139 strain SG24 and O1 El Tor strain N16961 (Table 1) were also used as positive and negative controls, respectively. Integration of the SXT element into chromosome I of the IDH07118 strain was confirmed by PCR amplification of the SXT element–chromosome region (*attR*) using the primers SXT jun-F/Prfcint-R (Table 2). PCR amplification of ARG conferring resistance to streptomycin (*strAB*) was done using the primers (i) SXT_strA_-F/SXT_strA_-R and (ii) SXT_strB_-F/SXT_strB_-R (Table 2). Genomic DNA of *V. cholerae* O139 strain SG24 and a non-O1/non-O139 environmental strain VCE232 were used as positive and negative controls, respectively. Integration of the SI region into the small chromosome of the IDH07118 strain was detected by PCR amplification of the left as well as the right SI element–chromosome junction using the primers SIjun-F1/SIjun-R1 and SIjun-F2/SIjun-R2, respectively. In the above PCR assays, genomic DNA of *V. cholerae* O1 El Tor strain N16961 and *E. coli* DH5α were used as positive and negative controls, respectively. For PCR amplification *Taq* DNA polymerase (Invitrogen, USA) was always used along with appropriate buffer as directed by the company. PCR assay was performed using the GeneAmp PCR system (Model 9700; Applied Biosystems, USA).

**Table 2.** Primers used in this study

Primers	Sequence (5′−3′)	Purpose	Reference
SXTint-F	GCTGGATAGGTTAAGGGCGG	PCR and sequencing	[49]
SXTint-R	CTCTATGGGCACTGTCCACATTG	PCR and sequencing	[49]
FloRfl-F	CGGAATTCCCATCCTGCGGGAGCG	PCR, cloning and sequencing	This study
FloRfl-R	AACTGCAGTCCAGCGCTTTCACAC	PCR, cloning and sequencing	This study
FloR-F	CCGGAATTCGTCATGACCACCACACGC	Cloning and sequencing	This study
FloRNΔ29-F	CCGGAATTCGCGATGGATATTTATCTCCCTG	Cloning and sequencing	This study
FloRCΔ36-R	AAAACTGCAGCTAGTCACCGTTTAAAAGTG	Cloning and sequencing	This study
PBAD-F	TCGCTAACCAAACCGGTAAC	Sequencing	[47]
PBAD-R	GATGCCTGGCAGTTCCCTAC	Sequencing	[47]
SXTjun-F	ATAAAGTCAAGATCAGCGAAAAT	PCR and sequencing	This study
Prfcint-R	AGAGTCAACTGCGGTCAGAG	PCR and sequencing	[49]
FloRcΔ12-R	AAAACTGCAGCTACCGAAGGAGCACCAGCCCC	Cloning and sequencing	This study
FloRnΔ4-F	CCGGAATTCACCATGCGCCCCGCGTGGGCCTATAC	Cloning and sequencing	This study
FloRP1-F	CCGGAATTCGTAGCAATTCATATTC	Cloning and sequencing	This study
FloRP2-F	CCGGAATTCTCATCTGATTGCTGACG	Cloning and sequencing	This study
SXT_strA_-F	GCGTGACCGCCTCATTTGG	PCR	This study
SXT_strA_-R	CCCGTGCATTGAAGAGTTTTAG	PCR	This study
SXT_strB_- F	ATGTTGCTCGAATATGCCGG	PCR	This study
SXT_strB_- R	GCCGGATCGTAGAACATATTGG	PCR	This study
SIint-F	ACATCCATTTTCATAAT	PCR	This study
SIint-R	GTGCATTTGGATACTTT	PCR	This study
SIjun-F1	ATTAAAGCTGGTCAATAC	PCR	This study
SIjun-R1	GAAGCTTACCTTCATTGGAT	PCR	This study
SIjun-F2	ATACAAAAAAGCTTCAGTTC	PCR	This study
SIjun-R2	GCTGCGCTGCTTCTAACT	PCR	This study
StrAB-F	CCGGAATTCCCATTGAATCGAACT	Cloning and sequencing	This study
StrAB-R	AAAACTGCAGGTCGCTTG	Cloning and sequencing	This study
StrA-R1	AAAACTGCAGGAACATCAACCCCAAGT	Cloning and sequencing	This study
StrB-F1	CCGGAATTCTTGATGTTCATGCCG	Cloning and sequencing	This study
StrA-F2	ATTGCTAACGCCGAAGAG	RT-PCR	This study
StrB-R2	CCGCGCAGTTCATCAGCAAT	RT-PCR	This study
StrAcΔ6-R	AAAACTGCAGCTACAATCGCAGATAGAAGG	Cloning and sequencing	This study
StrAnΔ27-F	CCGGAATTCGTTATGTTTCGACGTGGTGACG	Cloning and sequencing	This study
FloRrtm-F	TGTCGCGGTCGGTATTGTC	qRT-PCR	This study
FloRrtm-R	CGTCGAACTCTGCCAAAGC	qRT-PCR	This study
StrA_rtm_-F	GGGCAGCGCCAGATGA	qRT-PCR	This study
StrA_rtm_-R	GCTTCGATCCCCAATACATTG	qRT-PCR	This study
M13-F	GTAAAACGACGGCCAGT	Sequencing	NEB
M13-R	AACAGCTATGACCATG	Sequencing	NEB
recA-F	GCAATTTGGTAAAGGCTCCA	qRT-PCR	[27]
recA-R	GTTGTGCAGCAGCAATCAGT	qRT-PCR	[27]

### Semi-quantitative reverse transcriptase PCR (RT-PCR) assay

For RT-PCR assay, *V. cholerae* cells were grown in LB at an OD_600_ value of 1.5 followed by extraction of total cellular RNA using the TRI Reagent (Sigma-Aldrich, USA), essentially as described earlier [25–27]. Purity checking and quantification of the prepared RNA were performed spectrophotometrically. RT-PCR assay was performed for confirmation of co-transcription of *strA* and *strB* genes present in the integrated SXT element of *V. cholerae* IDH07118 strain using purified cellular RNA and the gene-specific internal primers StrA-F2/StrB-R2 (Table 2) using the Qiagen One Step RT-PCR kit as directed by the manufacturer (Qiagen, Germany). The PCR-amplified product was checked by agarose gel electrophoresis using appropriate molecular size markers. To confirm the absence of any contaminating DNA in prepared RNA samples, PCR assay of each sample was also performed with *Taq* DNA polymerase (Invitrogen). Lack of amplification in the absence of RT confirmed that the desired PCR product was generated solely from cDNA.

### Quantitative RT-PCR (qRT-PCR) assay

To study the expression of *floR* and *strA* genes in the *V. cholerae* IDH07118 strain, qRT-PCR assay was performed. For the qRT-PCR assay, total cellular RNA was prepared from bacterial cells grown in LB medium to an OD_600_ value of ~1.5, as mentioned above. Reactions were performed using the One Step SYBRPrimeScript RT-PCR kit II, essentially as described by the manufacturer (Takara Bio, Inc., Japan). qRT-PCR assay was performed using the CFX96 Real-Time System (Biorad, USA). The primer sets FloRrtm-F/FloRrtm-R and StrA_rtm_-F/StrA_rtm_-R (Table 2) were used for qRT-PCR analysis. Relative expression values (*R*) were calculated using the equation *R*=2^−(Δ*C*_T_target−*C*_T_reference)^, where *C*_T_ is the fractional threshold cycle. In each experiment as an internal control the *recA* gene-specific primers recA-F/recA-R (Table 2) were used. Each assay was repeated at least three times.

### Construction of plasmids

The recombinant plasmid pDFloR (Amp^r^; Table 1) was constructed by PCR amplification of the *floR* ORF with its natural promoter (size 1.9 kb) using the primers FloRfl-F/FloRfl-R (Table 2) and genomic DNA of the *V. cholerae* non-O1/non-O139 strain IDH07118 (Table 1) as a template followed by digestion with the enzymes *Eco*RI*/Pst*I, ligation of the fragment in similarly digested vector DNA pDrive using ligase enzyme (Promega, USA), the transformation of the ligation mixture in competent *E. coli* DH5α cells (Table 1) and plating on LA plate containing ampicillin. After incubation of the plate at 37 °C overnight, ampicillin-resistant clones were selected and checked for the presence of the desired recombinant plasmid. Among multiple clones obtained one clone was selected and named pDFloR (Amp^r^; Table 1). The recombinant plasmid pFloRORFBAD (Amp^r^; Table 1) was constructed by only PCR amplifying the ORF region (~1.4 kb) of the *floR* of IDH07118 using the primers FloR-F/FloRfl-R (Table 2) and genomic DNA of the *V. cholerae* non-O1/non-O139 strain IDH07118 (Table 1) as a template followed by digestion with the enzymes *Eco*RI*/Pst*I and cloning in similarly digested vector DNA pBAD24 (Amp^r^; Table 1). In the same manner, recombinant plasmids pStrABBAD, pStrABAD and pStrBBAD (Table 1) were constructed by PCR amplifying the *strAB* (~1.65 kb), *strA* (~0.8 kb) or *strB* (0.85 kb) ORF using different region-specific primers StrAB-F, StrAB-R, StrA-R1 and StrB-F1 (Table 2) and genomic DNA of the strain IDH07118 (Table 1) as a template followed by digestion of the PCR-amplified fragment with the enzymes *Eco*RI*/Pst*I and cloning in similarly digested vector DNA pBAD24 (Amp^r^; Table 1). For the construction of plasmids carrying the *floR* and *strA* ORFs with either N- or C-terminal coding region deletions, the desired fragments were PCR-amplified using different sets of primers, namely, FloRN∆29 F, FloRn∆4 F, FloRC∆36 R, FloRc∆12 R, StrAn∆27 F, StrAc∆6 R, etc. (Table 2) and using genomic DNA of the *V. cholerae* non-O1/non-O139 strain IDH07118 (Table 1) as a template followed by digestion of the desired fragments with *Eco*RI/*Pst*I and cloning in similarly digested vector DNA pBAD24 (Table 1). The 5′/3′-end-deleted *floR/strA*ORFs were amplified with forward primers carrying an artificially inserted ATG start codon or reverse primers carrying an artificially inserted TAG stop codon, respectively (Table 2). Other than this, the recombinant plasmids pDFloR∆P1 and pDFloR∆P1P2 (Table 1) were constructed by PCR-amplifying the *floR* ORF with or truncated promoter region (creating ~1.58 and ~1.47 kb PCR fragments) using region-specific primers (Table 2) and genomic DNA of the *V. cholerae* non-O1/non-O139 strain IDH07118 (Table 1) as a template followed by digestion with the enzymes *Eco*RI*/Pst*I and cloning of the fragment in similarly digested vector DNA pDrive (Table 1). The authenticity of each construct was always verified by restriction digestion and DNA sequencing (data not shown) using relevant primers (Table 2).

### Protein expression assay

For expression analysis, bacterial cells carrying recombinant pBAD24-derived plasmid expressing either the wild-type (WT) or the mutant alleles were induced with 0.2 % l-arabinose (Sigma-Aldrich, USA). Strain carrying the empty vector pBAD24 was used as a negative control. Whole-cell lysates of the strains were subjected to 12 % sodium dodecyl sulfate polyacrylamide gel electrophoresis (SDS-PAGE) essentially as described previously [28]. The molecular weights of protein bands were determined using standard molecular weight markers (NEB, USA).

### DNA sequencing and computational analyses

DNA sequencing was performed using the BigDye Terminator v3.1 Cycle Sequencing kit (Applied Biosystems, Inc., USA) essentially as instructed by the company. Samples were run on an ABI 3130 Genetic Analyzer (Applied Biosystems) using the POP-7 polymer (Applied Biosystems). DNA sequence data were compiled and analysed using Chromas 1.45 (http://www.technelysium.com.au/chromas.html).

The National Center for Biotechnology Information (NCBI) blastn and blastp programs were used to search for homologous nucleotide or protein sequences, respectively, in the database (www.ncbi.nlm.nih.gov). Deduced amino acid sequences were subsequently subjected to a database search using blastp (www.ncbi.nlm.nih.gov). clustal Omega software (www.ebi.ac.uk/Tools/msa/clustalo/) was used for the alignment of protein sequences and further modified through GeneDoc software version 2.7.000 (www.psc.edu/biomed/genedoc). GeneDoc were used for the alignment of DNA and protein sequences. In designing primers for PCR assay and other experiments, Primer3 software was used. TM domain prediction was performed using TMHMM Server v.2.0 (www.cbs.dtu.dk/services/TMHMM/). Apart from these programs, the following software was also used for: restriction site mapping (http://www.restrictionmapper.org/); promoter prediction(http://www.fruitfly.org/seq_tools/promoter.html,http://www.softberry.com/berry.phtml); primer analysis (http://insilico.ehu.es/PCR/) and operon information (http://www.biocyc.org/).

### Whole-genome sequencing and comparative genomics of O1 and non-O1/non-O139 *V. cholerae*

Three isolates (FGL9582, FGL6615, FGL7710), which were phenotypically either resistant or susceptible to antimicrobials, were subjected to whole-genome sequencing on an Illumina MiSeq platform as described earlier [29]. The raw reads were subjected to the trimmomatic sequence analysis tool (http://www.usadellab.org/cms/?page=trimmomatic), followed by *de novo* genome assembly (SPAdes, v3.1) using the raw reads with QC values of ≥30. The assemblies were looked for contamination and completeness using the CheckM tool [30]. In addition to the study isolates, whole-genome sequences deposited in the public domain were also included (Table S1, available in the online version of this article). A total of 8159 genome assemblies of all serogroups of *V. cholerae* available in the Enterobase database (dated as of 1 November 2022), were downloaded (http://enterobase.warwick.ac.uk/species/vcholeare/). These assemblies were screened through the dereplicator tool to remove closely related genomes (https://github.com/rrwick/Assembly-Dereplicator). A total of 486 representative genomes (with the addition of 3 study isolates) have been chosen with a threshold of 0.01 in the dereplicator tool, which was used for further analysis. The assemblies were annotated using a species-specific database in the prokka annotation tool [31]. The gff files generated in prokka were further used to capture the core genomes through the panaroo pipeline with default parameters [32]. Single-nucleotide polymorphisms (SNPs) in the core-genome alignment were extracted by SNP-sites scripts and a maximum-likelihood phylogenetic tree was generated with a GTR-GAMMA-based algorithm by RAxML tool [33]. The phylogenetic tree was annotated with metadata using iTOL web server [34]. In addition, *V. cholerae* genomes were sub-typed by the presence of markers such as *rfbV* and *wfbZ* genes that are specific for O1 and O139 serogroups, respectively, using a mapping-based approach through the Snippy tool (v.4.6.0) [35]. Genomes that did not possess either of these two genes were considered as non-O1/non-O139 serogroup. The presence of the *int* SXT element was screened using the integrase as a marker gene as earlier [36] by the Snippy tool. Further, the presence of acquired antimicrobial-resistant genes related to *int* SXT was screened through the ABRicate database (https://github.com/tseemann/abricate). Additionally**,** the genomes were also analysed for the typical characteristics of *V. cholerae* using MyDBFinder available at the Cholera Finder database in CGE (https://cge.food.dtu.dk/services/CholeraeFinder/). The genomes of the three *V. cholerae* strains were blasted against the ICE SXT reference sequence (GQ463141) using proksee to infer the genetic arrangements (https://proksee.ca/projects/new).

### Nucleotide sequence submission

Raw read fastq sequences of three *V. cholerae* strains were submitted to the NCBI Sequence Read Archive (SRA) database.

## Results

### Analysis of the SXT element in the genome of *V. cholerae* strain IDH07118

**Fig. 1.**
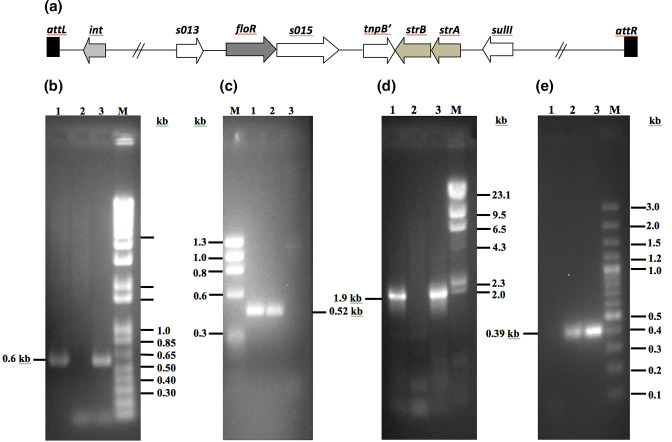
(**a**) Schematic representation of the genetic organization of the 5’-end of an SXT element (truncated region is shown). The *attL* and *attR* are the attachment sequences present in the 5’- and 3’-ends of the element. Arrows indicate different genes and their direction of transcription. Arrangements of the genes *int*, *floR* and *strAB* are as shown. (**b**) Confirmation of the presence of SXT element in *V. cholerae* non-O1/non-O139 strain IDH07118. Using region-specific primers, the left chromosome–SXT junction carrying the internal part of the *int* gene of ~0.6 kb in size was obtained by PCR amplification. Lanes: 1, *V. cholerae* SG24 (positive control); 2, *V. cholerae* N16961 (negative control); and 3, *V. cholerae* IDH07118. M indicates 1 kb DNA Ladder Plus (Invitrogen) used as a molecular size marker with sizes (in kb) indicated in the right margin. (**c**) Using region-specific primers, the right SXT–chromosome junction carrying the internal part of the *int* gene in IDH07118 of ~0.52 kb was obtained by PCR amplification. Lanes: 1, *V. cholerae* SG24 (positive control); 2, *V. cholerae* IDH07118; and 3, *V. cholerae* N16961 (negative control). M denotes φX174 DNA digested with *Hae*III used as a molecular size marker with fragment sizes (in kb) shown in the left margin. (**d**) Detection of the *floR* in *V. cholerae* IDH07118. Using the *floR*-specific primers, a ~1.9 kb DNA fragment was PCR-amplified. Lanes: 1, *V. cholerae* SG24 (positive control); 2, *V. cholerae* N16961 (negative control); and 3, *V. cholerae* IDH07118. M indicates λ DNA digested with *Hin*dIII used as a molecular size marker with sizes (in kb) indicated in the right margin. (**e**) Detection of the *strA* in *V. cholerae* IDH07118. Using the *strA*-specific primers, a ~0.39 kb DNA fragment was PCR-amplified. Lanes: 1, *V. cholerae* VCE232 (negative control); 2, *V. cholerae* SG24 (positive control); and 3, *V. cholerae* non-O1/non-O139 strain IDH07118. M indicates 100 bp Plus DNA Ladder (Thermo Scientific Generuler) used as a molecular size marker with sizes (in kb) indicated in the right margin.

*V. cholerae* IDH07118 (Table 1) is a non-O1/non-O139 clinical strain isolated from a diarrhoeal patient admitted to ID Hospital, Kolkata, India, and it showed resistance to Cam (10 µg ml^−1^) and Sm (200 µgml^−1^). However, it was not known whether the genome of IDH07118 carries the SXT element, which usually confers the Cam^r^ and Sm^r^ phenotypes. Therefore, to confirm the presence of the SXT element in the genome of IDH07118, we amplified the SXT region using a specific set of primers (Table 2). The SXT element-positive Cam^r^ and Sm^r^ *V. cholerae* O139 strain SG24 (Table 1) was used as a positive control [11]. *V. cholerae* O1 strain N16961 (Sm^r^; Table 1) and a non-O1/non-O139 strain VCE232 (Table 1) were used as negative controls wherever needed. The SXT element always carries the *int* gene (usually at its 5′-end) coding for the integrase enzyme because it is needed for chromosomal integration [9]. A schematic representation of the genetic organization of an SXT element is shown in Fig. 1a. To identify the presence of the *int* gene in the genome of IDH07118, the region-specific primers SXTint-F and SXTint-R (Table 2) were designed. When PCR assay was carried out with these primers using the genomic DNA of the IDH07118 strain as templates, the desired PCR amplicon of ~600 bp in size was obtained (Fig. 1b). As expected, while the genomic DNA of the SXT element negative strain N16961 did not give an amplicon, the genomic DNA of *V. cholerae* SG24 strain used as a positive control gave a ~600 bp amplicon using the same primer set. Thus, the result supports the view that the SXT element is present in the genome of the non-O1/non-O139 strain IDH07118. It has been reported that the SXT element integrates into the 5′-end of the *prfC* present in the large chromosome of *V. cholerae* [10]. Thus, a region-specific PCR assay was performed to determine the integration site of the SXT element into the chromosome of IDH07118, which yielded an expected PCR amplicon of 527 bp (Fig. 1c) using the primers SXTjun-F/Prfcint-R (Table 2). For further confirmation, the 600 bp (carrying the probable *int* genic part of the SXT element) and 527 bp (carrying the right junction of the SXT-*prfC* genic region) PCR amplicons were subjected to nucleotide sequencing. Each sequence showed 99 % identity with the existing GenBank sequences for this genetic element, which confirmed the presence of the SXT element in the large chromosome of the *V. cholerae* non-O1/non-O139 strain IDH07118. Since the SXT element carries the chloramphenicol and streptomycin resistance genes (*floR* and *strA*, respectively), the result prompted us to check for the presence of these genes in strain IDH07118. For this, a PCR assay was performed using the primers FloRfl-F/FloRfl-R (*floR*-specific; Table 2) or SXT_strA_-F/SXT_strA_-R (*strA*-specific; Table 2) and the genomic DNA of IDH07118 strain as templates. As expected, the desired amplicon of ~1.9 kb was obtained using the FloRfl-F/FloRfl-R primers, while the SXT-negative strain N16961 yielded no amplicon (Fig. 1d). Similarly, for the *strA*-specific PCR assay yielded the desired amplicon of ~395 bp, while the genomic DNA of the *V. cholerae* VCE232 strains used as a negative control yielded no amplicon (Fig. 1e).

### Molecular characterization of the *floR* and *strA* genes of the SXT element of IDH07118

In this study, the entire *floR* including its promoter region of the strain IDH07118 was amplified by PCR, and cloned in the pDrive vector. The recombinant clone thus obtained was named pDFloR (Table 1). To characterize further, the *floR*, the insert DNA of the pDFloR, was subjected to nucleotide sequencing followed by analysis. Analysis of the sequence revealed that the ORF of the *floR* is ~1.22 kb long. Nucleotide blast analysis showed about 99 % similarity with the *floR* of *E. coli, Acinetobacter baumannii* and *Bordetella bronchiseptica*, further confirming that the *V. cholerae* IDH07118 strain indeed carries the *floR* gene. The *floR* gene of IDH07118 strain codes for a 404 amino acid protein, which also showed 99 % identity with other known FloR sequences deposited in GenBank. Similarly, the *strAB* of the SXT element present in the genome of the *V. cholerae* IDH07118 strain were studied by cloning each of the ORFs of the *strA* and *strB* genes separately and also *strAB* together in the expression plasmid pBAD24 (Table 1). The clones obtained were designated pStrABAD, pStrBBAD and pStrABBAD, respectively (see details of these clones in Table 1). The insert DNA of the clones were checked by sequencing followed by blast analysis and then subjected to phenotypic assays using sensitive bacterial cells. Nucleotide blast analysis of the sequence revealed that the *V. cholerae* strain IDH07118 indeed carries both *strAB* as an operon and showed ~99 % similarity with the *strAB* genes of *V. cholerae* O1 El Tor, *E. coli, K. pneumoniae* and *A. baumannii*. The analysis also revealed that the *strA* codes for a 268 amino acid long protein and showed 99 % identity with other known StrA sequences deposited in GenBank.

### Identification of the *floR* promoter region

**Fig. 2.**
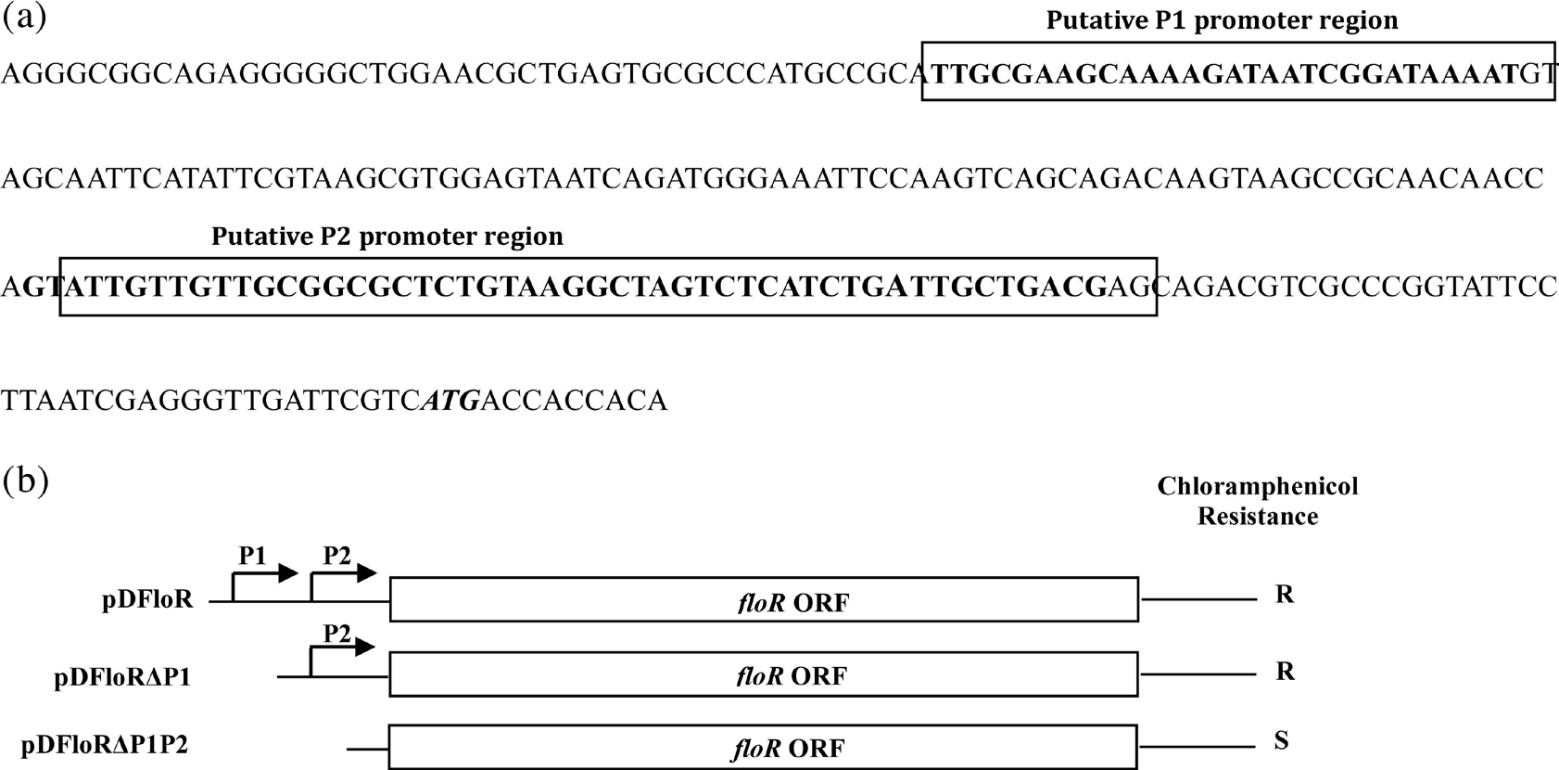
(**a**) Nucleotide sequences of two putative promoters, P1 and P2, of the *floR* gene of the *V. cholerae* strain IDH07118. Predicted P1 and P2 regions are as shown in boxes. The start codon of the *floR* gene is in bold and italics. (**b**) Schematic diagram (not drawn to scale) showing predicted promoters (bent arrows), P1 and P2, with the *floR* ORF. Three recombinant plasmids, pDFloR, pDFloRΔP1 and pDFloRΔP1P2, were constructed, which carry P1+P2, only P2 and no P1 and P2, respectively, as shown. Chloramphenicol-sensitive *E. coli* or *V. cholerae* strains carrying each of these plasmids showing chloramphenicol resistance/sensitivity patterns are also indicated in the right margin.

Since no information is currently available about the regulation of expression of the *floR*, we attempted to characterize its promoter region. Ideally, the most common class of bacterial promoters, including those of housekeeping genes, carry the upstream consensus sequences at the −10 and −35 regions, which are 5′-TATAAT-3′ and 5′-TTGACA-3′, respectively. These conserved sequences are critical interaction sites for the housekeeping sigma factor σ^70^. Hence, bioinformatics analysis of the upstream region of the *floR* was carried out using BDGP promoter prediction software (http://www.fruitfly.org/seq_tools/promoter.html and http://www.softberry.com/ berry.phtml), which predicted two putative promoter regions, called P1 and P2, as shown in Fig. 2a. Therefore, experiments were designed to identify the active P*_floR_* by deletion analysis of the predicted promoter sequences and the approach taken is shown schematically in Fig. 2b. When the recombinant construct pDFloR (Amp^r^; Table 1) carrying the putative promoter region of the *floR* was introduced into a chloramphenicol-sensitive *V. cholerae* or *E. coli* strain, it provided chloramphenicol resistance, suggesting that any or both the promoters are active. This success in assaying the promoter activity of the *floR* led to the design of the truncated version of the region to understand which one of the two putative promoters, P1 or P2, is critical. For this, recombinant plasmids pDFloRΔP1 and pDFloRΔP1P2 were constructed (see details in Table 1). When each of these constructs was tested in a sensitive *E. coli* DH5α or *V. cholerae* N16961 strain, only pDFloRΔP1 provided resistance to chloramphenicol and pDFloRΔP1P2 failed to do so. Thus, the result strongly suggests that P2 is most likely the active promoter responsible for the expression of the *floR*. We believe that the P1 promoter activates conditionally upon encountering a specific signal from the environment. However, dissecting the promoters and assessing them with a promoterless vector could be a possibility to determine the possible role of P1 promoter in *floR* transcription.

### Expression of the *floR* under different growth phases

**Fig. 3.**
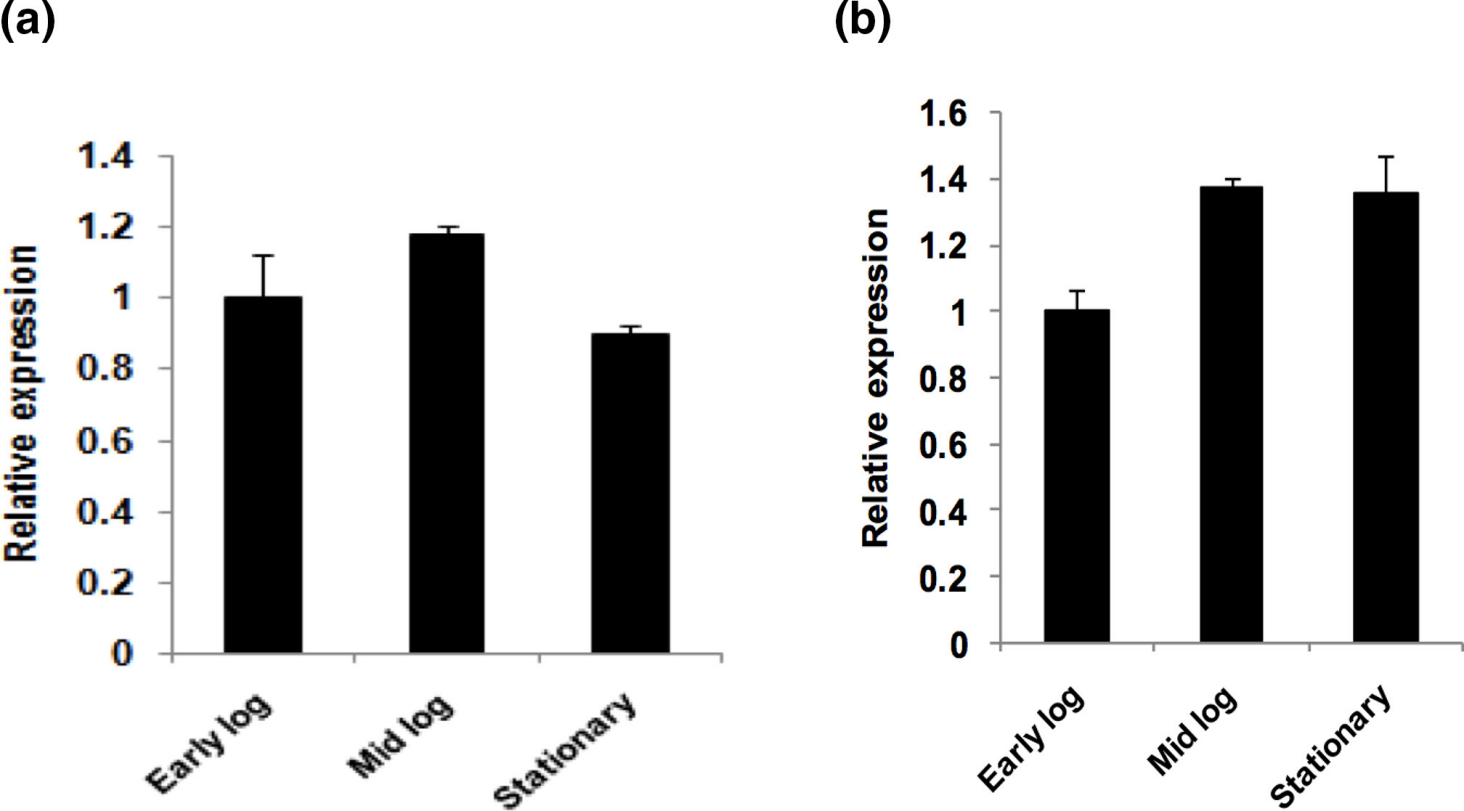
Relative expression of the (**a**) *floR* and (**b**) *strA* genes in *V. cholerae* strain IDH07118. qRT-PCR assays were carried out with total cellular RNA isolated from *V. cholerae* cells at three different growth phases in LB medium, early log (OD_600_=~0.5), mid log (OD_600_=~1.0) and stationary (OD_600_=~1.5), as shown. Each set of experiments was repeated at least thrice.

At present, little is known about the importance of expression of antibiotic resistance genes. Therefore, an attempt was made to understand the expression status of the *floR* in *V. cholerae* IDH07118 under different growth phases. For this, the total cellular RNA of bacterial cells growing at different growth phases was isolated followed by qRT-PCR analysis. Such analysis indicated that the *floR* was expressed during all growth phases and it is thus constitutive in nature, as shown in Fig. 3a.

### Validation of transcriptional organization of the *strAB* genes and its expressional study

**Fig. 4.**
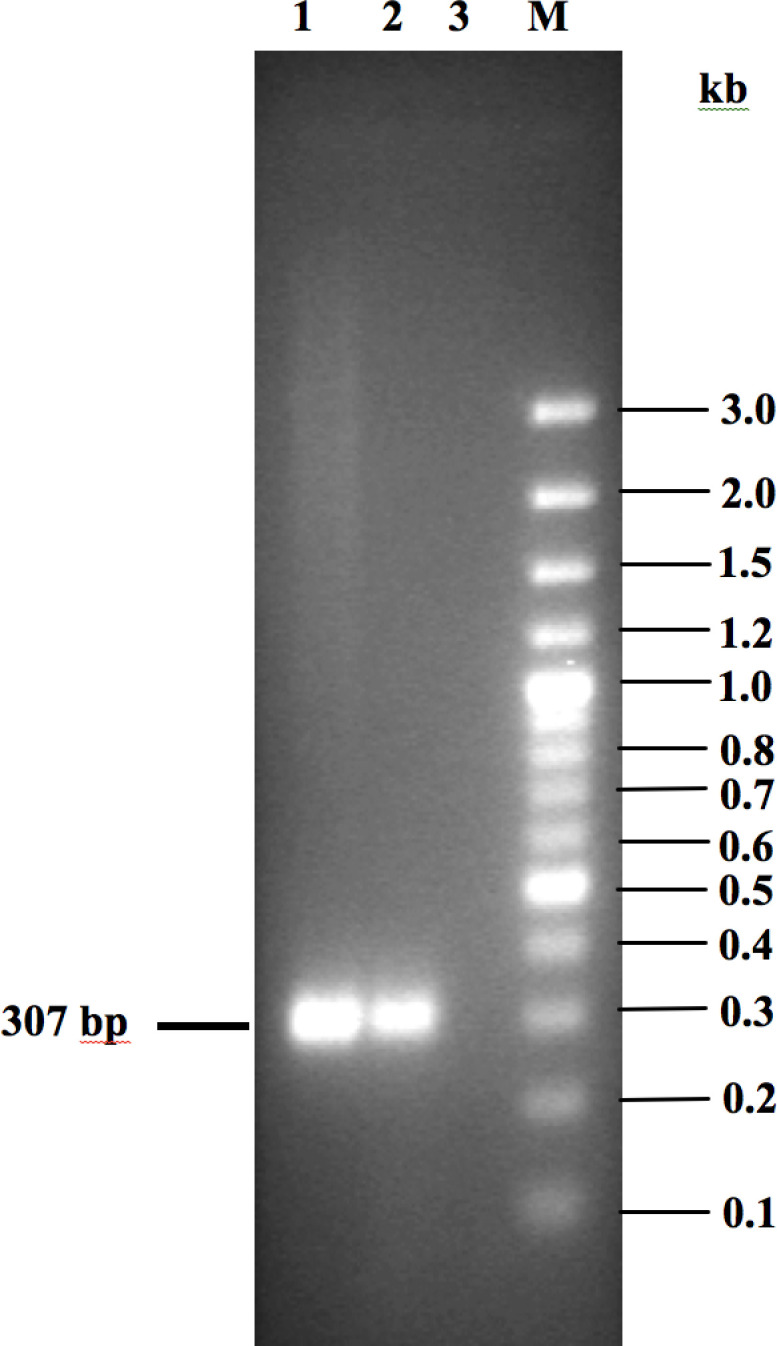
Semi-quantitative RT-PCR assay to verify transcription of *strA* and *strB* genes as a polycistronic mRNA from the operon. PCR amplification of the junction of the *strA* and *strB* genes yielded an expected PCR amplicon ~307 bp in size. M denotes 100 bp Plus DNA Ladder (Thermo Scientific) and the fragment sizes (in kb) are shown in the right margin. Lanes: 1, genomic DNA of *V. cholerae* non-O1/non-O139 strain IDH07118 gDNA (positive control); 2, stationary phase RNA of *V. cholerae* non-O1/non-O139 IDH07118 with reverse transcriptase and *Taq* DNA polymerase; and 3, stationary phase RNA of *V. cholerae* non-O1/non-O139 IDH07118 with *Taq* DNA polymerase only (negative control).

Bioinformatics analysis indicated that the *strAB* genes are transcribed as a bicistronic operon. To confirm whether *strAB* is indeed co-transcribed as an operon, internal primers StrA-F2/StrB-R2 (Table 2) were designed from upstream and downstream regions of *strAB* followed by semi-quantitative RT-PCR assay with total cellular RNA isolated from the stationary phase (OD_600_ = ~1.5) culture of the *V. cholerae* non-O1/non-O139 strain IDH07118. As expected, a desired ~0.3 kb amplicon was obtained when the primer set StrA-F2/StrB-R2 (Table 2) was used and this confirmed that the *strAB* genes are indeed present in an operon (Fig. 4). A similar result was obtained when the stationary phase RNA of the strain *V. cholerae* SG24 was used as a positive control. Next, to understand the transcriptional status of the *strAB* operon, qRT-PCR analysis was performed using only the *strA*-specific primers (Table 2) and bacterial RNA prepared from cells at different phases of growth. Like the *floR*, expression of the *strAB* operon also appeared to be constitutive in nature in the *V. cholerae* strain IDH07118, as shown in Fig. 3b. Here the *V. cholerae* O139 strain SG24 was also used as a positive control, and showed similar results.

### Mutational analysis of the *floR* and *strA* genes

**Fig. 5.**
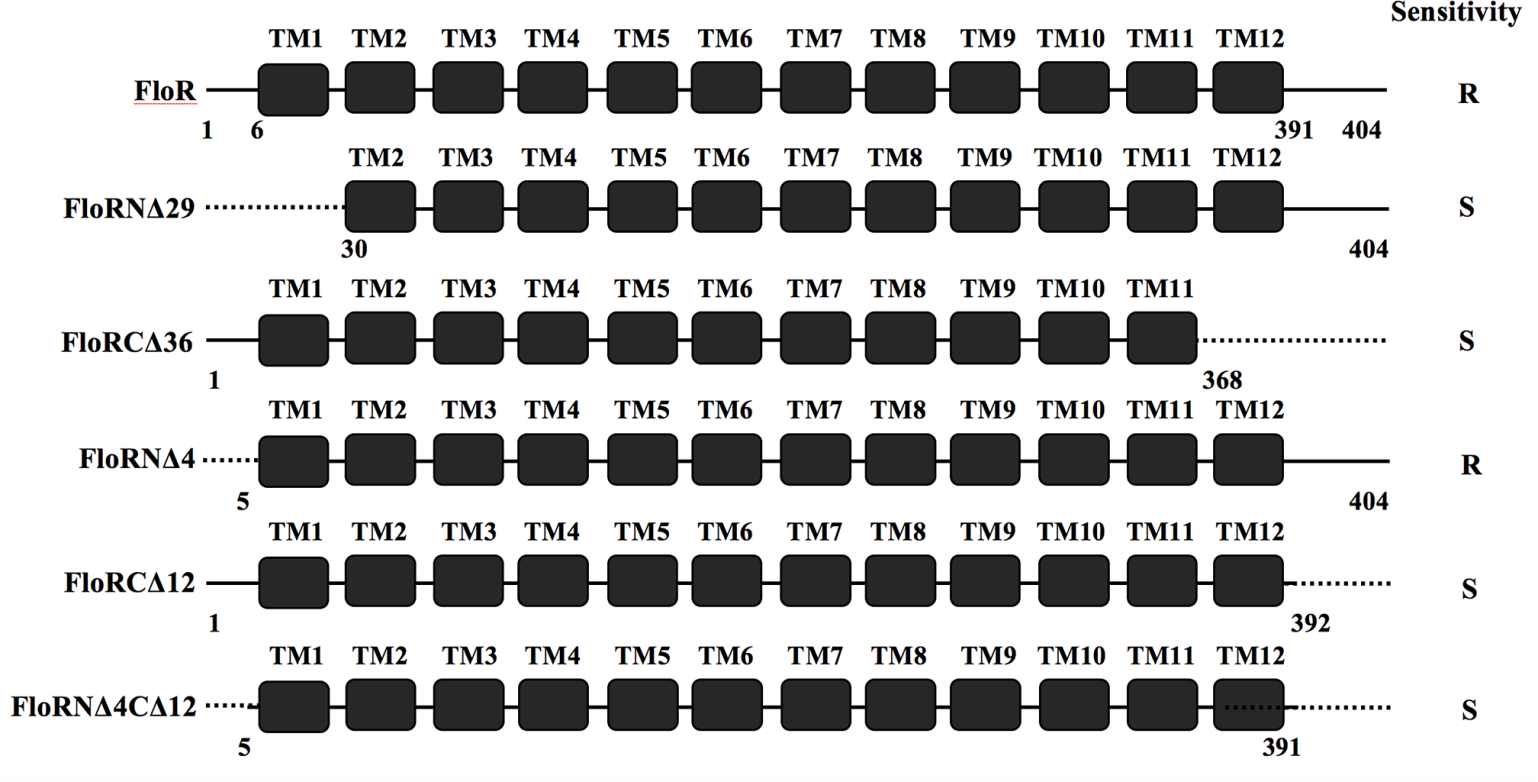
Schematic diagram (not drawn to scale) showing the WT and N- and C-terminal-deleted mutant proteins of FloR. WT FloR is 404 amino acids long. Mutant protein alleles FloRN∆29, FloRC∆36, FloRN∆4, FloRC∆12 and FloRN∆4C∆12 generated in this study are indicated in the left margin. TM1 to TM12 indicate transmembrane domains that are present within 6 to 391 amino acid sequences of the FloR protein, as indicated. Thin lines indicate intactness of the protein sequence in each case and dotted lines indicate deleted portions of the protein. Chloramphenicol resistance of the WT or any mutant allele when expressed through the recombinant plasmid in chloramphenicol sensitive strain is indicated in the right margin as R (resistant) or S (sensitive).

After establishing that the expression of the *floR* gene *in trans* in chloramphenicol-sensitive strains leads to development of resistance against the antibiotic, a mutational approach was taken to characterize the functionality of the FloR. To do this, only the ORF of the *floR* gene was cloned under the arabinose inducible promoter (P*_BAD_*) of the plasmid pBAD24, designated pFloRORFBAD (Amp^r^; Table 1) and the functionality of the ORF present in the clone was checked by introducing the plasmid pFloRORFBAD in chloramphenicol-sensitive *V. cholerae* strain N16961 followed by induction of the FloR expression in the presence of l-arabinose (see Methods section) and growing N16961 (pFloRORFBAD) in the presence of chloramphenicol. As expected, the strain N16961 (pFloRORFBAD) showed resistance to chloramphenicol, whereas bacteria carrying the empty vector pBAD24 [N16961(pBAD24)] showed sensitivity towards the antibiotic. After establishing this, two constructs were further designed where either the N- or the C-terminal coding region of the *floR* gene was deleted (Fig. 5), and each of these constructs was again introduced separately into chloramphenicol-sensitive *V. cholerae* strain N16961 as described above. Since the FloR carries 12 TMDs (TMD1 to TMD12), in 1 construct, called pFloRNΔ29BAD (Amp^r^; Table 1), 29 amino acids of the N-terminal region of FloR carrying the TM1 of the coding region of the *floR* gene were deleted. Similarly, in another construct, named pFloRCΔ36BAD (Amp^r^; Table 1), 36 amino acids of the C-terminal region of FloR carrying the TM12 of the coding region were deleted (Fig. 5). When each of these constructs was introduced separately in chloramphenicol-sensitive *V. cholerae* N16961 cells, both of them failed to provide resistance, which strongly suggests that deletion of even a single TMD region either from N- or C-terminal is indeed critical for the functioning of the FloR. Similarly, three other deletion constructs were made, where either the N- or the C-terminal coding region or both were deleted (Fig. 5). When each of these constructs was introduced separately into chloramphenicol-sensitive *V. cholerae* N16961 strain, the plasmid pFloRCΔ12BAD (where the 12 amino acid coding region of the C-terminal end of FloR was deleted), pFloRNΔ4BAD (where the 4 amino acid coding region from the N-terminal end was deleted) or pFloRNΔ4CΔ12BAD (where 4 and 12 amino acid coding regions of the N- and C-terminal ends, respectively, were deleted) (see Table 1), only the recombinant plasmid pFloRNΔ4BAD when introduced in chloramphenicol-sensitive *V. cholerae* or *E. coli* cells was able to provide resistance against chloramphenicol, whereas pFloRCΔ12BAD or pFloRNΔ4CΔ12BAD plasmid carrying strains remained susceptible to this antibiotic. Thus, the results strongly suggest that apart from N- and C-terminal TMDs, C-terminal amino acids beyond these domains are also critical for the functioning of the FloR. This is the first report that shows that C-terminal end amino acids are critical for FloR activity.

**Fig. 6.**
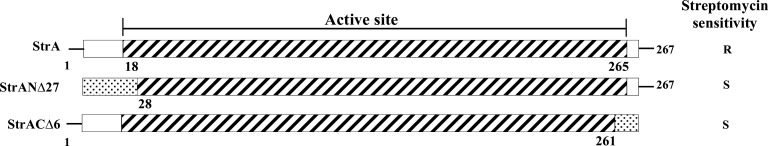
Schematic diagram (not drawn to scale) showing the WT and N- and C-terminal-deleted mutant proteins of StrA. The WT StrA protein is 267 amino acids long, as shown. Striped regions represent active sites of the StrA protein. Deleted N- or C-terminal regions of StrA are indicated by dotted boxes. Streptomycin resistance was conferred by the WT but not by the StrA mutant allele StrAN∆27 or StrAC∆6 when expressed through the respective recombinant plasmid in streptomycin-sensitive strain, as indicated in the right margin, R,resistant; S, sensitive.

**Fig. 7.**
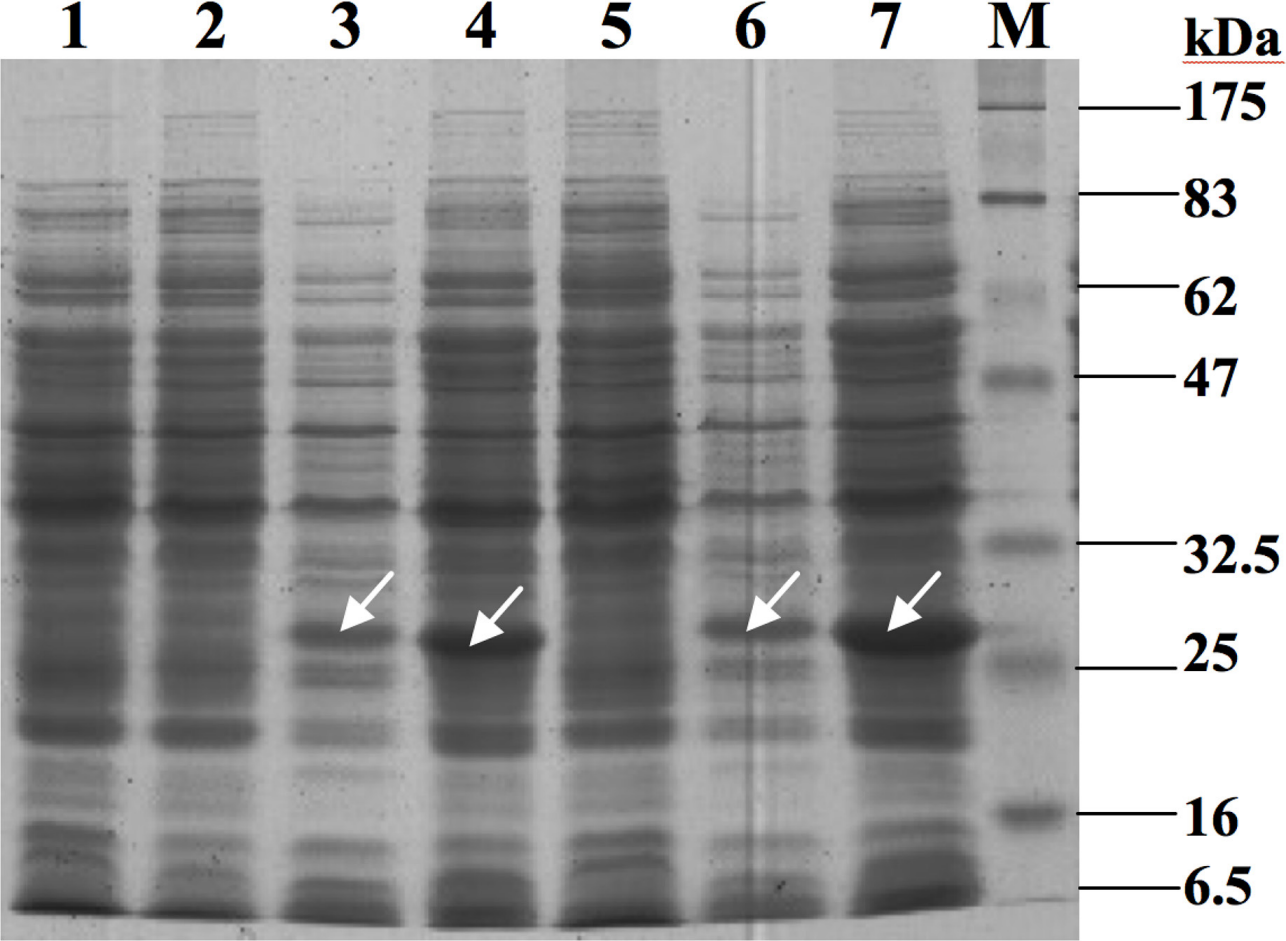
Expression analysis of the StrAB and StrA proteins. Each *V. cholerae* strain was grown for 6 or 16 h in LB medium with or without 0.2 % arabinose followed by preparation of bacterial cell lysates and 12 % SDS-PAGE analysis. Lanes: 1, VCE232(pBAD24); 2, VCE232(pStrABBAD) without arabinose induction; 3, VCE232(pStrABBAD) with arabinose induction for 6 h; 4, VCE232(pStrABBAD) with arabinose induction for 16 h; 5, VCE232(pStrABAD) without arabinose induction; 6, VCE232(pStrABAD) with arabinose induction for 6 h; 7, VCE232(pStrABAD) with arabinose induction for 16 h. White arrows indicate expressed protein bands. M denotes prestained broad range protein markers (6–175 kDa).

After establishing that the expression of the *strAB in trans* in streptomycin-sensitive *V. cholerae* VCE232 strain leads to the development of resistance against the antibiotic, a mutational approach was employed to further functionally characterize the StrA. When the nucleotide sequence of *strA* was analysed through the Conserved Domain Database (CDD, NCBI), the result indicated that the product of the gene belongs to the aminoglycoside-3′-phosphotransferase subfamily, which itself is part of a larger superfamily that include 3 catalytic sites of other kinases, namely (i) an active site containing 30 amino acid residues, (ii) an ATP-binding site of 15 amino acid residues that are overlapped with the residues of the active site and (iii) an antibiotic-binding site that contains 12 amino acid residues. Thus, to further characterize the *strA* gene, two constructs, pStrANΔ27BAD and pStrACΔ6BAD (Table 1), were made, where either the N- or the C-terminal coding region was deleted (Fig. 6). In the deletion construct pStrANΔ27BAD (Table 1), the N-terminal 27 amino acids, including 3 residues of ATP-binding site coding region, were deleted. In the deletion construct pStrACΔ6BAD (Table 1), the C-terminal six amino acids, including three residues for the antibiotic-binding site coding region, were removed. When each of these constructs was introduced separately into streptomycin-sensitive *V. cholerae* VCE232 or *E. coli* DH5α strains, they failed to confer streptomycin resistance. The results strongly suggest that both N- and C-terminal amino acid sequences are critical for the functioning of the StrA protein (Fig. 6). Apart from mutational analysis we also tried to see the expression of the StrAB proteins by SDS-PAGE analysis. To do this, the strains VCE232(pStrABBAD) and VCE232(pStrABAD) were grown overnight at 37 °C in the presence or absence of 0.2 % arabinose (as an inducer of gene expression), followed by whole-cell lysate preparation and SDS-PAGE analysis of the lysates. A streptomycin-sensitive VCE232 strain carrying the empty vector pBAD24 [VCE232(pBAD24)] was used as a control. Analysis of the SDS-PAGE indicated expression of the desired StrAB and StrA proteins under the conditions tested, as shown in Fig. 7.

### Comparative genomic analysis of *V. cholerae* isolates in a global context

**Fig. 8.**
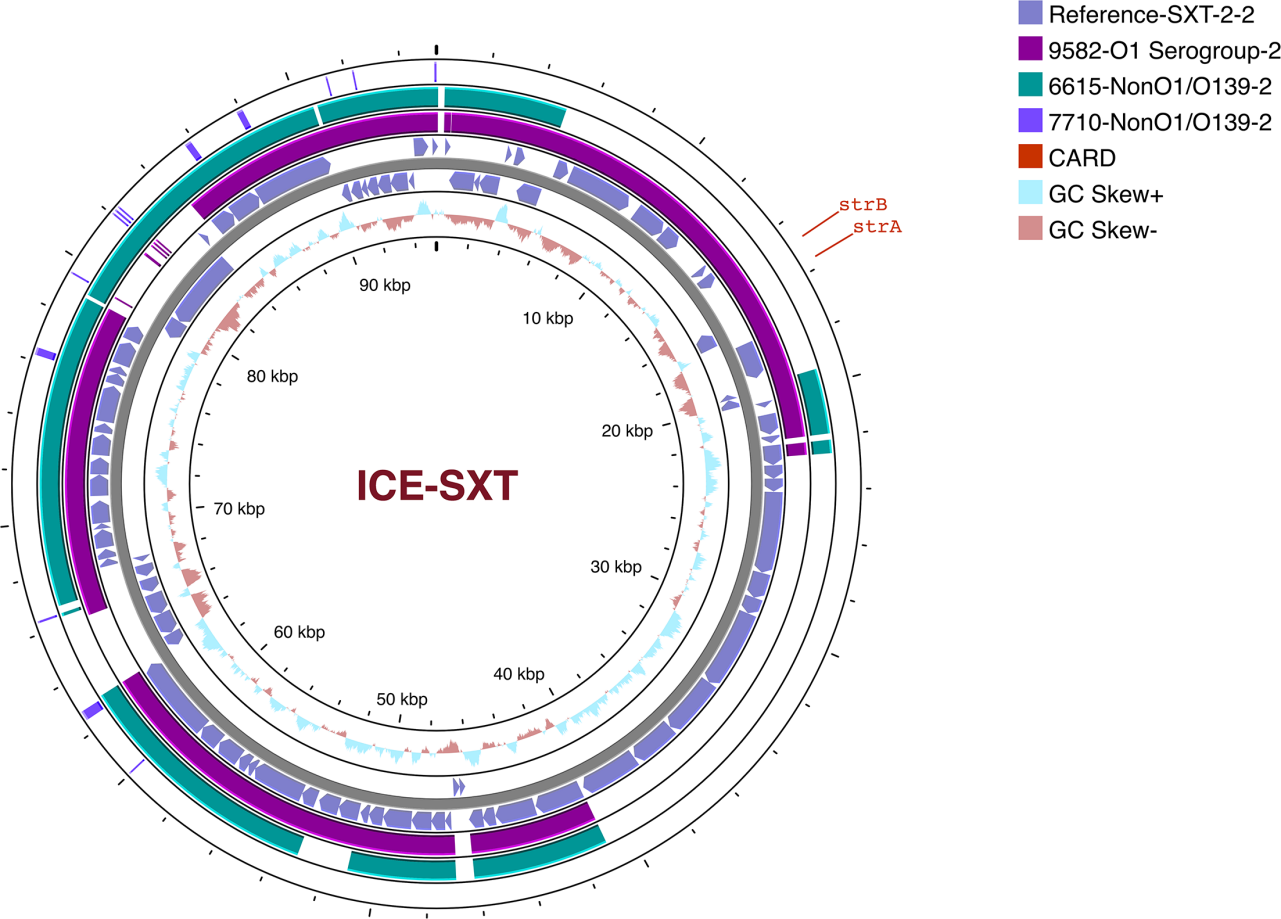
blast analysis to infer the genetic backbone of the ICE-SXT element harbouring AMR gene cassettes in *V. cholerae*. Arrows indicate the orientation of open reading frames in the reference genome of *V. cholerae*. The colour key represents the *V. cholerae* strains sequenced in this study.

**Fig. 9.**
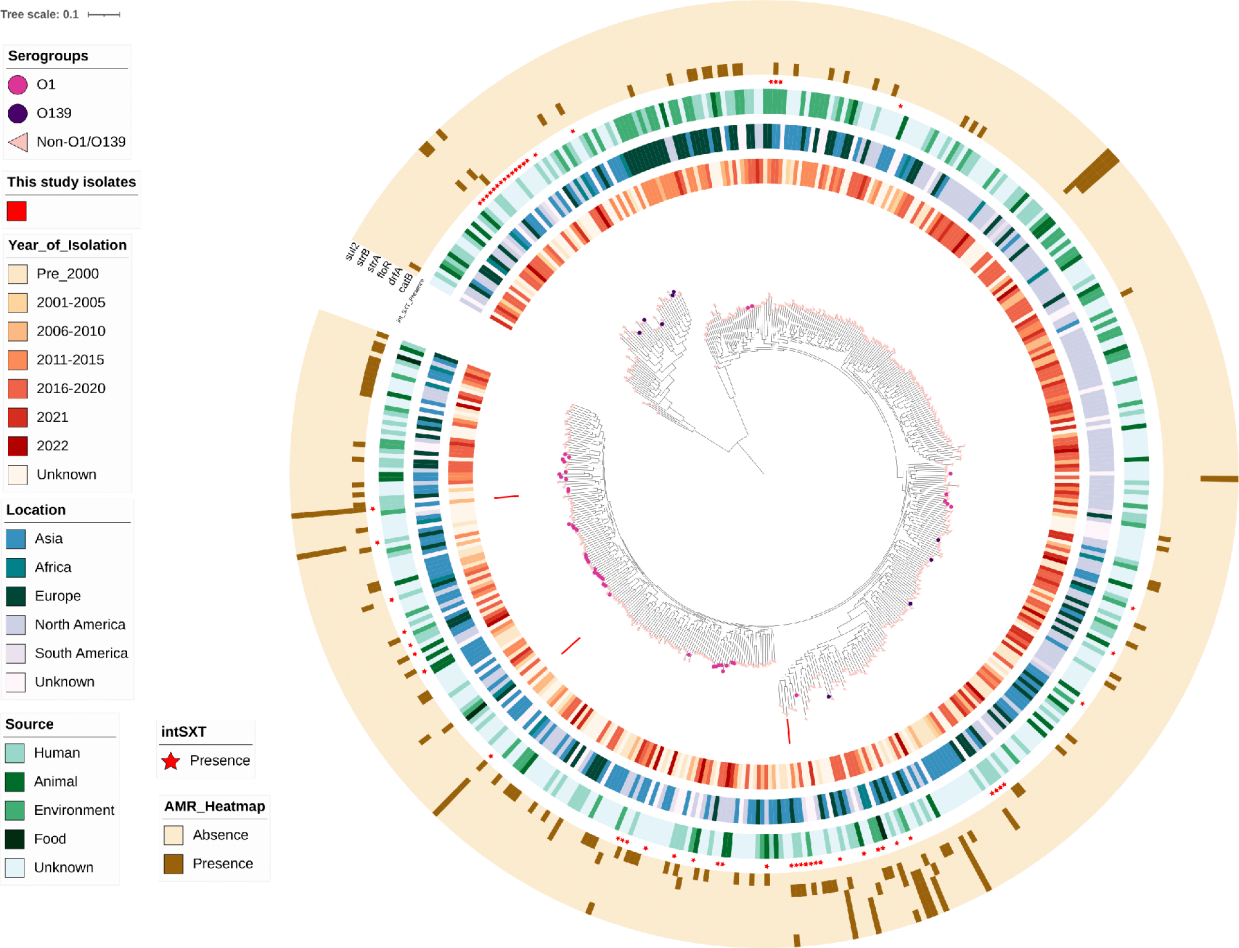
Maximum-likelihood phylogenetic tree based on SNPs from 2695 core genes of 489 *V. cholerae* genomes. Branch symbols represent the serogroups of *V. cholerae.* Red line indicates *V. cholerae* isolates used in this study, colour strips represent the location, year and source of isolation of *V. cholerae* strains. The tree scale indicates the substitution rate per genome per year.

The genomic characteristics of the three *V. cholerae* strains sequenced in this study are shown in Table 3. These strains were identified as serogroups of O1 (FGL9582) and non-O1/non-O139 (FGL6615, FGL7710). The O1 strain (FGL9582) was typed as belonging to the 7PET late-wave-3 strain that carried the CtxB7 toxin type with a multidrug resistance profile harbouring six AMR genes (*strA, strB, floR, catB9, dfrA1* and *sul2*), and it also contained the ICE-SXT element. Additionally, it also harboured pathogenicity islands such as VSP-1 and 2 and VPI-1 and 2 that carry genes encoding for virulence. In contrast, the non-O1/non-O139 strains showed a distinct profile, in which strain FGL6615 harboured *dfrA*15. Interestingly, *dfrA15* was found to be associated with the presence of only one integrase of a class 1 integron family adjacent to it within the same contig. The presence of one of the VPI-2 genes, VPI-2_VC1790, that encodes for the transposase gene was detected; however, the query coverage of the gene was lower (70/345 bp). It was also interesting to note that 6615_S37 was found to contain 57 % of the ICE-SXT backbone, but the AMR cassettes region was completely absent (Fig. 8). Another non-O1/non-O139 strain, FGL7710, was lacking both the AMR genes and ICE elements; however several genes belonging to VPI (*n=2*) and VSP2 (*n=8*) islands were present. Of note is the presence of genes that encode for phage integrase in both the VPI and VSP2. All three study isolates harboured multiple virulence genes, such as *als, hlyA, makA, rtxA* and *toxR,* besides the strain-specific genes (9582_S34: *VgrG*, *ompT*, *ace*, *zot*, *mshA*, *ompU*, *vasX*, *ctxA*; 7710_S36: *OmpU*, *mshA*, *vspD*; 6615_S37: *VasX*). Global phylogeographical analysis has been performed to determine the positioning of the study isolates in the global *V. cholerae* species tree (Fig. 9). A core-genome-based SNP tree identified the genetic relatedness of *V. cholerae* genomes of serogroups O1, O139 and non-O1/non-O139. Non-O1/non-O139 genomes were placed distantly in the phylogenetic tree despite their similar year of isolation. Certain serogroup-specific clusters of O1 were identified, but no clear discrimination between O1, O139 and non-O1/non-O139 serogroups could be made, as they were dispersed throughout the tree, irrespective of the source and year of isolation (Fig. 9). The O1 strain (FGL9582), which displayed a multidrug resistance profile, was found to harbour an ICE-SXT element, identified by both the CGE database and by the Snippy tool. Among the two non-O1/non-O139 strains, FGL6615 harboured an integrase gene, but it only carried the *dfrA* gene; whereas FGL7710 harboured neither integrase nor any AMR gene. The association of the presence of integrase with the AMR genes was not comparable, as there was a mixed profile, hence no significant comparisons could be made. Isolates with the profile of integrase gene-positive did not harbour their associated AMR genes and vice versa. Such a diverse profile was observed irrespective of the sample collection type being of clinical or environmental origin.

**Table 3.** Information concerning the *V. cholerae* genomes sequenced in this study

*V. cholerae* strains	FGL9582	FGL6615	FGL7710
Serogroup	O1	Non-O1/non-O139	Non-O1/non-O139
Sequence size (bp)	4 042 309	3 943 885	4 115 476
Number of contigs	92	152	106
GC content (%)	47.5	47.6	47.5
Shortest contig size	128	128	128
Median sequence size	1197	733	1260
Mean sequence size	43 938.1	25 946.6	38 825.2
Longest contig size	632 677	369 825	719 115
N50 value	247 188	246 904	359 640
L50 value	5	7	4

## Discussion

Owing to the use of chloramphenicol, streptomycin and sulfamethoxazole/trimethoprim, antimicrobial pressure has subsequently been attributed to the acquisition of SXT-ICE encoding multiple AMR-encoding gene cassettes. The SXT element appears to be important in the evolution of *V. cholerae* O1 and O139 serovars. Abrupt loss of the ICE-SXT element with a gene cassette containing *floR*, *strAB*, *sul2* and *dfrA18* was found to be one of the reasons for the epidemiological decline of the O139 serogroup [12].

In this study, we have examined a non-O1/non-O139 clinical strain IDH07118 of *V. cholerae*, which showed resistance to more than one antibiotic, i.e. chloramphenicol and streptomycin, due to the presence of the SXT element. We confirmed the presence of the SXT element by mapping the *int* and the junctions of its integration. In SXT elements of *V. cholerae*, the arrangement and the composition of the antibiotic resistance gene clusters differs among various serogroups and in many geographical locations [37, 38]. For e.g. the SXT elements of various *V. cholerae* O1 strains carrying *strAB*, *sulII*, *floR* and other antibiotic resistance genes [22, 37, 39], which are not reported in non-O1/non-O139 serogroups. Some of the *V. cholerae* strains with SXT element and antibiotic resistance genes may not carry the *floR* [22, 40]. Carraro *et al.* [41] have reported a novel mobilizable genomic island, which integrated into the 3′ end of the *trmE* gene in the large chromosome of a *V. cholerae* non-O1/non-O139 strain carrying different antibiotic resistance genes like *floR, tetA, blaP, sulI,* etc. Karlsson *et al.* [22] have shown intermediate susceptibility to chloramphenicol by *V. cholerae* O1 strains carrying the *floR*, which could be due to its low expression. Our data suggest that the *floR* product is responsible for efflux of the chloramphenicol antibiotic in *V. cholerae.* Chloramphenicol resistance due to other mechanism rather than enzymatic inactivation has been reported [16, 42]. The presence of *floR* that codes for an efflux protein of group E3 was found to be responsible for resistance against chloramphenicol [9, 43]. It should be noted that for the first time we functionally characterized the *floR* in *V. cholerae*. Molecular and functional characterization of FloR helped in the identification of critical domains/amino acid sequences at the N- and C-terminal regions, which are responsible for the chloramphenicol efflux activity. Bioinformatics analysis of the FloR with a transmembrane prediction tool suggested the presence of 12 TMDs, as described previously [21, 44]. The presence of the *floR* in various Gram-negative bacteria, including in the SXT element of *V. cholerae*, has also been reported [9, 45]. We tested the drug specificity of FloR both in *V. cholerae* as well as in *E. coli* background. Prediction of the natural promoter region for the chloramphenicol resistance gene *floR* was made and the putative promoter sequences P1 and P2 have been identified. Further, genetic and mutational analysis indicated that the P2 promoter region is essential for the expression of the *floR*. All of the genetic constructs were functionally validated using the chloramphenicol-sensitive *V. cholerae* or *E. coli* strains. For this, the ORF of *floR* and its different truncated forms were introduced in both DH5α and C6709 strains, and evaluated the capacity of the resulting construct conferring drug resistance to the host cell. Mutations were constructed by deleting the 12th TM and then progressive deletion of 10th, 11th and 12th TMs from the C-terminal region, followed by first TM deletion from the N-terminal region. This selection was made because they belong to the extreme N-terminal and C-terminals of the 12 TM regions. To support the above data, antimicrobial susceptibility testing has been performed with deletion constructs in comparison with ORF construct of *floR*. Loss of chloramphenicol resistance character was observed in all deletion constructs, whereas ORF construct of *floR* maintain its chloramphenicol resistance. Other than TM domains, amino acids of the C-terminal domain also seem to be important to provide chloramphenicol resistance. These data indicate that the involvement of every single TMD is important in the efflux of chloramphenicol. Nucleotide blast analysis of the sequence revealed that the strain IDH07118 indeed carries the *floR* and it showed ~99 % similarity with the *floR* of *E. coli, A. baumannii* and *B. bronchiseptica*. Thus, *floR* is a highly conserved gene with a 404 amino acid long protein that has 99 % identity with other known FloR sequences deposited in the GenBank database. Structural analysis of FloR revealed extensive similarity to other FloR found in other Gram-negative bacteria. Expressional analysis of the *floR* was performed using qRT-PCR assay and it was observed that the *floR* is a constitutively expressed gene in *V. cholerae* non-O1/non-O139 strain IDH07118 and this is the first report about growth-specific expression of the *floR*. Analysis of the *strAB* operon present in the SXT region of the non-O1/non-O139 strain IDH07118 indicated that they are most likely expressed through a single promoter and the polycistronic expression of the genes was confirmed in the RT-PCR assay. For functional analysis, *strAB* or *strA* were cloned under the arabinose inducible promoter P*_BAD_* of the expression vector pBAD24 and induction of expression by arabinose in streptomycin-sensitive *V. cholerae* or *E. coli* strain conferred resistance. This assay further helped in establishing the functional domains/regions of these proteins by deletion analysis. qRT-PCR analysis for the expression of *strA* indicated that this is also a constitutively expressed gene in the *V. cholerae* non-O1/non-O139 strain IDH07118 since it could be detected in all the growth phases of the cells. Mutational analysis of the *strA* gene using several plasmid constructs indicated that deletion of 27 amino acids from the N-terminus or 6 amino acid residues from the C-terminal end of the StrA leads to functional loss of the protein. Expression of StrA was further tested by SDS-PAGE analysis and it was found that the StrA protein is expressed within 6 h of incubation of bacterial cells and it is quite stable since huge accumulation occurs even after 16 h of incubation of bacterial cells.

Core-genome-based phylogenetic analysis of *V. cholerae* could not discriminate the strains of respective serogroups. This could probably be due to the conserved core genomes of *V. cholerae* species, as the genes encoding for O-antigen-determining serogroups were not included in the core-genome and hence the tree could not distinguish the respective serogroups. However, the identification of ICE-SXT in the non-O1/non-O139 irrespective of the serogroups/sample origin further signifies the diversity of these elements. This has previously been reported in Thailand, with non-O1/non-O139 environmental and clinical strains harbouring closely related ICE-SXT elements that carry AMR genes [46].

In summary, to our knowledge, this is the first report of the presence of the SXT element, which integrates into the 5′ end of *prfC* containing *floR* and *strA* genes, with its extensive molecular and functional characterization of a non-O1/non-O139 clinical *V. cholerae* strain. In addition, this is the first report showing the importance of the N- and C-terminal amino acid residues for the enzymatic activity of the StrA.

## Supplementary Data

Supplementary material 1
